# Knowledge, Perceptions, Attitudes and Practices of Midwives Regarding Maternal Influenza and Pertussis Vaccination: A Qualitative Study

**DOI:** 10.3390/ijerph19148391

**Published:** 2022-07-09

**Authors:** Antonia Arreciado Marañón, María Isabel Fernández-Cano, Laura Montero-Pons, Maria Feijoo-Cid, Azahara Reyes-Lacalle, Rosa María Cabedo-Ferreiro, Josep Maria Manresa-Domínguez, Gemma Falguera-Puig

**Affiliations:** 1 Department of Nursing, Faculty of Medicine, Campus Bellaterra, Universitat Autònoma de Barcelona, 08193 Barcelona, Spain; antonia.arreciado@uab.cat (A.A.M.); maria.feijoo@uab.cat (M.F.-C.); jmanresa@idiapjgol.info (J.M.M.-D.); 2 Multidisciplinary Research Group in Health and Society (GREMSAS), 08007 Barcelona, Spain; 3 Atenció a la Salut Sexual i Reproductiva de Santa Coloma de Gramenet, Direcció d’Atenció Primària Metropolitana Nord, Institut Català de la Salut, 08921 Barcelona, Spain; lmontero.ics@gencat.cat; 4 Research Group Atenció a la Salut Sexual i Reproductiva (GRASSIR), Institut Universitari d’Investigació en Atenció Primària Jordi Gol (IDIAP Jordi Gol), 08007 Barcelona, Spain; areyesl.ics@gencat.cat (A.R.-L.); rcabedo.ics@gencat.cat (R.M.C.-F.); gfalguera.mn.ics@gencat.cat (G.F.-P.); 5 Atenció a la Salut Sexual i Reproductiva de Sabadell, Direcció d’Atenció Primària Metropolitana Nord, Institut Català de la Salut, 08203 Barcelona, Spain; 6 Atenció a la Salut Sexual i Reproductiva de Granollers, Direcció d’Atenció Primària Metropolitana Nord, Institut Català de la Salut, 08400 Barcelona, Spain; 7 Unitat de Suport a la Recerca Metropolitana Nord, Institut Universitari d’Investigació en Atenció Primària Jordi Gol (IDIAPJGol), 08303 Barcelona, Spain; 8 Atenció a la Salut Sexual i Reproductiva Metropolitana Nord, Direcció d’Atenció Primària Metropolitana Nord, Institut Català de la Salut, 08911 Barcelona, Spain

**Keywords:** influenza, immunization, midwives, pertussis, pregnancy, vaccination

## Abstract

The coverage of maternal vaccination against pertussis and, particularly, influenza is lower than expected. The lack of recommendation from healthcare providers conditions non-vaccination in pregnant women. The purpose was to determine the knowledge, perceptions, attitudes and practices of midwives regarding maternal influenza and pertussis vaccination. A qualitative descriptive study based on semi-structured, face-to-face interviews with seventeen midwives was conducted, including purposive sampling and thematic analyses. Midwives had disparate knowledge and perceptions about the severity of influenza and pertussis in pregnant women, and influenza was not considered very serious. The vaccines were generally considered safe. However, because midwives did not have enough information about the safety of the influenza vaccine, there was a tendency not to recommend it. While most midwives had a positive attitude toward vaccination, their advocation for vaccination against influenza was not as clear as it was for pertussis. Not wanting to influence the decision and assuming an informative–facilitating role also led providers to recommend the influenza vaccine less frequently. Midwives are among the main sources of professional advice for pregnant women. Addressing their understanding and professional practices regarding maternal vaccination is key to change the attitude of pregnant women and thus increase vaccine uptake among them, particularly for influenza.

## 1. Introduction

Maternal vaccination against influenza and pertussis protects the mother from these infections and any related complications, as well as the fetus during the first months of life while awaiting vaccination [1,2,3,4,5,6,7]. It is considered an effective and safe strategy [6,8,9,10] and is recommended by various benchmark institutions [1,2,5,11]. The Advisory Committee on Immunization Practices (ACIP) recommends the influenza vaccine for all women at any stage of their pregnancy during the influenza season [12], as well as the tetanus toxoid, reduced diphtheria toxoid and acellular pertussis vaccine (Tdap) during each pregnancy between weeks 27 and 36, although preferably at the start of this period [13]. Despite this recommendation, maternal vaccination coverage is lower than expected [8,14], particularly for influenza. In the United States, 56.6% of pregnant women received the Tdap vaccine, and 61.2% received the influenza vaccine (2019–2020 season) [15]. The latter represents a moderate increase over previous seasons (53%, 2016–2017 season) [16]. In Australia, vaccination coverage was 65–80% for pertussis and 35–60% for influenza [17]; in Flanders, Belgium, it was 64% and 45%, respectively (2014–2015 season) [18]; in Spain, the vaccination rates were 83.6% (year of 2019) and 50% (2019–2020 season), respectively [19].

The factors that influence maternal vaccination vary depending on the context and population [20]. Demographic and clinical factors related to low maternal vaccination coverage include age, low socioeconomic and educational level, belonging to an ethnic minority, single marital status or not living with a partner, previous preterm birth, no or few prenatal visits and no health insurance [21]. The factors reported by pregnant women as influential in their decision to get vaccinated are being informed, the influence of others and having access to vaccines [22,23], although the availability of these does not always guarantee vaccine uptake [24]. Of all the factors, a recommendation from healthcare providers (HCPs) [14,25,26] and more specifically midwives [27] stands out as the most important. In this sense, the main barrier to vaccination is the lack of recommendations from HCPs and aspects linked to safety [14,25,26]. The fact that some women claim they would have been vaccinated had their HCPs recommended it [22,27] demonstrates the vital role that providers play in maternal vaccination. For their part, HCPs have reported that inadequate training, reimbursement issues, concerns about safety, increased workload and having staff available with the certification required to administer vaccines [14,20,28] are the main barriers to recommending vaccination.

Maternal care providers (MCPs) are the teams responsible for the monitoring and follow-up of pregnant women. Depending on the context, these teams may comprise exclusively obstetricians–gynecologists or also include general practitioners and midwives. They play a crucial role in providing information and making recommendations to pregnant women; therefore, they play a crucial role in improving vaccination coverage [28]. According to MCPs, the main barriers to recommending maternal vaccination include a deficit of knowledge about vaccines and concerns about their efficacy and safety [21]. The literature shows that these barriers are greater among midwives than obstetricians–gynecologists [29]. In Spain, midwives are responsible for the monitoring and follow-up of healthy pregnant women, representing their main source of information [30]. They are also in charge of administering vaccines. In Spain, vaccines are administered free of charge by the public health system and can be accessed from primary care centers and sexual and reproductive care units (ASSIR; from the Spanish *Atención a la Salud Sexual y Reproductiva*). Therefore, midwives play a key role in maternal vaccination. However, there is still much to understand about their knowledge, perceptions, attitudes and practices in this regard. Collecting extensive and comprehensive information about the phenomenon using a qualitative methodology that offers the perspective of the midwives could offer a better understanding thereof.

The aim of this paper is to determine the knowledge, perceptions, attitudes and practices of midwives regarding maternal influenza and pertussis vaccination. Their knowledge can contribute to improving clinical practice, resulting in increased vaccination coverage in pregnant women.

## 2. Materials and Methods

### 2.1. Design

This was a qualitative descriptive study. Qualitative studies are used as an in-depth approach to research phenomena that are difficult to quantify numerically, including feelings, interpretations, meanings and behavioral aspects [31]. Qualitative data can shed light on the complexity of human behavior [32], which is why they were appropriate in this study to understand practices related to maternal vaccination from the perspective of midwives and their experiences. On the other hand, qualitative description is an excellent methodological choice because it provides rich descriptive content from the subjects’ perspective [33]. This is achieved through a combination of sampling, data collection, analyses and techniques [34].

### 2.2. Setting and Participants

The study was conducted in a healthcare district of Barcelona Metropolitan Area (Catalonia, Spain) pertaining to the Spanish Public Health System, where some 7700 professionals work [35]. The healthcare district has seven ASSIR units, where pregnant women receive monitoring and follow-up from midwives, obstetricians–gynecologists, psychologists and clerical workers.

The study population comprised midwives working at ASSIR units in the aforementioned district. The inclusion criteria were to be eager to participate and to have at least six months of experience working as a midwife. In Spain, midwives are nurses who have completed a four-year university degree, followed by two years of specialized training. We used purposive sampling with a maximum variation strategy. The ultimate goal of purposive sampling is to obtain data-rich cases for the purpose of the study. Maximum variation sampling is used to capture a wide range of perspectives related to the phenomenon under study [34]. We thus included varied profiles of midwives to obtain maximum discourse variability, including midwives from ASSIR centers of different socioeconomic profiles; of different ages and years of professional experience as a midwife; with and without children; who had been vaccinated against the flu in the current campaign and/or previous ones and others who had not.

To access the population, assistance was provided by a leading midwife who was familiar with the ASSIR centers and identified potential participants after being informed about the study aim and different profiles required. From these potential participants, the first author made the final selection based on the required criteria. In the end, it was not necessary to contact all of them. Later, the leading midwife contacted each of the selected midwives via institutional email and invited them to participate in the study. After agreeing, interviews were scheduled for a day on which both the interviewer and the participants were available, at the end of their work shift, in a room at their workplace. At the time of the interview, the first author once again informed participants about the study and obtained their written informed consent. None of the selected midwives refused to participate.

### 2.3. Data Collection

Data were collected from November 2018 to February 2019 by means of semi-structured individual face-to-face interviews. This data collection technique is designed to obtain subjective responses from participants about a particular phenomenon that they have experienced [36]. In this study, that was the experience of midwives with recommending maternal vaccination. In the interaction between the interviewer and the informant in semi-structured interviews, the interviewee seeks to transfer his/her world to the interviewer, shedding light on his/her own meanings [37]. The semi-structured interview script [38] was written based on existing literature and deliberation by the investigators, who identified topics of interest based on the research aim (Table 1). Comprehensibility was tested by a midwife who did not participate in the interviews. Open-ended probing questions were used, as well as follow-up questions depending on how participants answered. A worksheet prepared by the investigators was used to gather data about the midwives, including sociodemographic data, interview data (conditions and development) and a summary of the interviewer’s first impressions upon completion of the interview.

The first author (female; Ph.D.) conducted the interviews. Additional investigators participated in three of the interviews by observing, taking notes and collaborating in the following deliberation. All the interviews were conducted at the participating midwives’ ASSIR units and were audio-recorded. Once transcribed, a member check was performed, sending each participant their transcription for review with the option to make changes. One midwife qualified some of the details of her responses. Another asked for her interview to not be used so it was removed from the study. None of the interviews had to be repeated. Data collection was finalized when theoretical saturation [39] was reached or, rather, when no new information was obtained from the interviews. This part of the process was discussed by the research team.

### 2.4. Data Analysis

A thematic analysis [37] was performed using ATLAS.ti 8. The following steps were taken: (1) Verbatim transcription. (2) Initial reading and rereading of the data until familiar with it. Creation of a list of tentative topics based on this pre-analysis. (3) Identification of text fragments that were meaningful for our research study (meaning units) and their coding. (4) Grouping of codes and construction of subcategories. Some subcategories were defined a priori based on the existing literature, research needs and team deliberation, while others emerged from the discourse. (5) Development of main categories, encompassing all the subcategories described. (6) Analysis of each category. These steps were all initially carried out by the first author who, later during each phase, contrasted, discussed and deliberated with another investigator until reaching consensus. At this point, the explanatory framework was developed alongside the existing literature. The other investigators participated in later deliberation.

### 2.5. Rigor

To ensure the rigor of the study, Lincoln and Guba’s [40] trustworthiness criteria were applied. All participants were given the opportunity to confirm or correct the information collected (member checking), thus ensuring credibility. Moreover, the analysis was first carried out individually and later contrasted with another researcher until reaching consensus. This last aspect, alongside the description of each phase of the process and the decisions taken, also helped achieve dependability. Transferability was guaranteed through the description of the context, the participants and selection procedure, the data collection and the analysis process. Lastly, the ongoing deliberation by the team during the research study and decision-making process favored confirmability. The research team was formed by nurses devoted to university teaching (Ph.D.) and practicing midwife nurses who also conduct research and management (also Ph.D.). Some of these nurses worked at the same healthcare center as some of the participants. However, the teaching researchers, including the first author (interviewer), and the participants did not previously know each other.

### 2.6. Ethics

This study was approved by the Primary Care Clinical Research Ethics Committee. Written informed consent was obtained from participants and an alphanumeric code was assigned to each participant to guarantee data confidentiality. The data were stored in password-protected computer files provided by the university and safeguarded by the first author.

## 3. Results

A total of 17 midwives participated in the study. Their ages ranged from 25–65 years. One midwife was excluded from the study at her request after reviewing the transcription of her interview and not feeling she had adequately expressed her philosophy. Therefore, the results correspond to 16 interviews, the characteristics of which can be found in Table 2. The mean duration of the interviews was 40 min (20–53). The results are presented by category and subcategory.

### 3.1. Knowledge and Perceptions about Influenza and Pertussis Infection and Vaccination in Pregnant Women

#### 3.1.1. Knowledge and Perceptions about Influenza and Pertussis Infections in Pregnant Women

For participating midwives, the risk pregnant women face of contracting influenza is similar to that of the general population or greater in the case of immune disorders that increase susceptibility, making them a risk group due to more numerous and severe complications. This severity and increased risk of complications mainly affect the mother at the respiratory level. While the midwives also consider how the fetus, newborn or course of the pregnancy might be affected, these aspects are mentioned less frequently and seem to be a repercussion.

‘The immune status of pregnant women is different and therefore influenza attacks it more. It’s the mother who may experience respiratory problems, which can lead to complications. Even death’.

(LL11)

Some admitted to minimizing the risk of contracting influenza, trivializing it as a common cold, and others, in some specific cases, attributed no complications to having influenza while pregnant.

‘I don’t think it’s an illness that causes—At least I can’t think of many examples of pregnant women who have experienced major complications from influenza. We have had, I work at the hospital, some women admitted with other viral infections’.

(LL07)

Some midwives expressed that they were not aware of concrete scientific evidence on influenza and its complications in pregnant women. Even so, they trust the healthcare institutions and the information they received on the topic.

‘What I have heard, even though I haven’t read the studies, I mean I don’t know their validity, they say there is a greater risk of a caesarean section, greater risk of premature birth, underweight babies. [...] Well, that’s what the studies say, right? It’s what the Catalan Government says, I imagine they must base it on studies they’ve done’.

(LL14)

As for pertussis, participants perceived it as being very severe, mostly and most severely affecting the newborn, as well as a high risk particularly during the first months of life. Although they mentioned potential respiratory issues in the mother, they attributed less importance and fewer complications to it. Most participants touched on the fact that the newborn’s immune system is not yet developed during the first months of life before the first vaccine doses. In any case, participants offered more knowledge about the complications of pertussis than of influenza.

‘It’s serious. Yes, yes, it’s serious and for the baby, from 0 to 2 months, it’s very, very serious’.

(LL04)

‘Pertussis is a disease, I tell women, that we’re all vaccinated against, that affects the respiratory system, but in a newborn it can be very serious. [...] It was decided that women should be vaccinated, but not for their own health, but so that the antibodies can be transferred to the baby and when he/she is born, during those two months until vaccination, he/she is more protected. That doesn’t mean the baby can’t get pertussis, but if he/she does, it won’t be as severe’.

(LL11)

When comparing the severity of pertussis with other diseases, participants placed it at a similar level, but higher than influenza.

‘But tetanus and pertussis are two significant diseases that can have more serious consequences than influenza.’

(LL01)

#### 3.1.2. Knowledge and Perceptions about Maternal Influenza and Pertussis Vaccination

Most participants considered the influenza and pertussis vaccines safe, without risks or with acceptable, minimal risks. This was sometimes clearly confirmed, without subtleties.

‘Very safe. They don’t affect the fetus, don’t affect it, they are proven—They are extremely safe—That is, there are prior studies, it’s not like saying “now we’re going to try this and see how it goes”. No. These vaccines do not affect the pregnancy at all. On the contrary, they protect you’.

(LL09)

Other midwives justified the safety of these vaccines based on the vaccination recommendations from institutions or their inclusion in the maternal vaccination schedule. If institutions recommended vaccination, participants understood that it was because there were studies supporting it and because it was safe to do so.

‘It’s safe. I mean, if it’s in the schedule, I expect there to be no risks. No doubt about it. No doubt’.

(LL13)

In other cases, participants questioned the safety of vaccines due to the harmful substances they allegedly contain. They mentioned that the very health institutions that recommended vaccination did not have information about this aspect. This created a professional conflict for midwives since they understood that they had to recommend the vaccine even while having doubts about its safety. This conflict sometimes had an impact on their recommendation of vaccines.

‘The safety of the vaccines is actually being called into question a bit. There are doubts about the mercury content, about—Well, I don’t know. I don’t know who evaluates the safety, but, as professionals, we don’t receive a lot of information. That is, we receive information like: “The Catalan Health System recommends X vaccine”. But we don’t receive much information about the safety of these vaccines. Really, we’re recommending something that could have adverse effects. [...] I mean, we work in the system and so, we follow the rules of the system, right? But I don’t know, I feel conflicted sometimes’.

(LL07)

‘I, well, ... Until they’re 100% guaranteed to have no side effects, I’m not sure I will recommend them, those that aren’t strictly necessary’.

(LL01)

While there were doubts about the safety of both vaccines, the pertussis vaccine was perceived as safer.

‘I think the pertussis vaccine is a bit safer, but safety isn’t really the issue, rather that the illness can be much more severe than influenza’.

(LL01)

The perception of missing information was not limited to safety. Some professionals reported knowing little about the vaccines, not being able to offer information beyond the institutional recommendation. Since they were not familiar with concrete scientific studies that supported such information they were not able to recommend the vaccines with more justification.

‘The truth is I don’t know much about it. Because I haven’t inquired much. They recommend that we vaccinate pregnant women. I justify it based on the recommendation from the—From the Catalan Government, to vaccinate them. But if they asked me for more information, I haven’t looked up many studies or anything to support whether they should get vaccinated or not’.

(LL15)

As for efficacy, the midwives expressed differences between the two vaccines. In the case of influenza, while some clearly confirmed the vaccine was effective, they spoke of relative efficacy depending on the virus mutation, and they mentioned doubts, having seen cases of influenza following vaccination. However, in the case of pertussis, there was a more generalized view of vaccine efficacy.

‘I don’t know if it’s effective (influenza vaccine). I don’t know. Some women, well, just as it happens with other people too, right? They say, “oh, after getting vaccinated I got a cold”’.

(LL12)

‘Yes, in fact, I also know there is scientific evidence showing a reduction in cases of newborns that have contracted pertussis since mothers have been vaccinated. Therefore, I understand that it is effective to administer the vaccine’.

(LL10)

The midwives also expressed varying ideas regarding the benefits of the vaccines. The influenza vaccine was understood to be beneficial primarily for the pregnant woman and secondarily for the newborn. Nevertheless, while the pertussis vaccine clearly benefitted the baby, it also favored the mother by preventing any guilt if she were to contract the illness due to not being vaccinated. The midwives did not apply this same benefit to the influenza vaccination.

‘I also think there’s an additional aspect with the pertussis issue, which is that—The guilt that the mother might feel when the baby is born and gets pertussis and she didn’t get vaccinated. That’s another factor to take into account, I think’.

(LL10)

### 3.2. Attitudes towards Maternal Influenza and Pertussis Vaccination

#### 3.2.1. Justification of Vaccination in Pregnant Women

Some participants believed maternal influenza and pertussis vaccination is justified. The argument was similar for both illnesses; the vaccines can prevent problems and complications and, while they are infrequent, it is important to avoid them. Moreover, the vaccines are not harmful to the woman, the course of pregnancy or the future baby. Therefore, there was no doubt that they should be recommended.

‘There could be a case in which nothing worse than influenza symptoms occur, but it’s also true that there have been severe cases. There haven’t been many, but when it has happened it has been severe enough to justify influenza vaccination. [...] There also haven’t been many cases of pertussis, but the cases that have taken place in the first two months are severe enough to justify vaccination’.

(LL8)

However, various midwives shifted the focus of this general justification. They sometimes hesitated to justify the influenza vaccine due to doubts about the frequency and severity of the illness. When assessing the risk of contracting pertussis and its severity, though, they found vaccination necessary and justified, even when there have been few cases of it, unlike influenza.

‘Hmm. I’m not certain. With the influenza vaccine, no. I don’t know if it’s justified. Because I also don’t know the data. [...] So, I don’t know if this is all justified, if the women with influenza had to stay home longer or if there were complications with pneumonia and issues which all justify vaccinating women. I’m not certain’.

(LL15)

‘I think it’s justified (pertussis vaccination), since, if there’s a way to prevent it that isn’t harmful to the mother, that isn’t harmful to the baby, then I think we have to do it’.

(LL14)

None of the midwives questioned the pertussis vaccination. There was only one doubt about the systematization of vaccines, which also pertained to the influenza vaccine. These participants understood that vaccination recommendations should be customized to each pregnant woman depending on her existing risks and circumstances.

‘This is always how it goes. Something happens, it gets established (the vaccination) and then—Sometimes you also have to know how to remove things that aren’t serving us. So, I don’t know if systematic vaccination is justified. I mean, what I’m questioning is the systematic nature of the vaccines’.

(LL07)

#### 3.2.2. Midwives’ Attitude toward Their Own Vaccination against Influenza

Most participating midwives were not vaccinated against influenza in the current campaign (4 of 16 were vaccinated) or previous campaigns (7 of 16 were vaccinated at some point). They expressed a variety of reasons for not being vaccinated, making special mention of never having had influenza, a lack of exposure to the virus and low perceived risk of becoming infected. They considered working with pregnant women in primary care a healthy and controlled environment that did not justify vaccination. For some, the risk of healthcare providers infecting their patients was considered a potential driving force for vaccination. However, they did not always perceive pregnant women as a risk group, unlike other patient groups.

‘I think that if I were a nurse for adults I might get vaccinated. It’s not very common in pregnant women. The patients we see are not—If they’re ill, it’s unlikely they would come to our office, you know? And pregnant women, well, of course, there are pregnant women who might come and spread something, but you don’t feel so exposed’.

(LL12)

‘I got vaccinated (against influenza) when I was at a NICU, so that I wouldn’t be an infectious agent. [...] It was very clear to me that I had to get vaccinated. But here I don’t see the need’.

(LL15)

Other reasons for not being vaccinated included the mild consequences of influenza if contracted, a desire to avoid taking foreign substances and a lack of information. In this case, participants referred to the need for specific evidence that would lead them to get vaccinated.

‘No. I haven’t been vaccinated against influenza. [...] To avoid putting more substances in my body’.

(LL04)

‘Maybe, if I had known more about the risks of not being vaccinated and the complications it might have for the mother and baby, maybe I would have been vaccinated. But I might not have knowledge about that’.

(LL10)

The midwives who did get vaccinated against influenza in the current or previous seasons justified doing so with the possibility of transmitting the virus themselves as professionals to pregnant women, who they considered a risk group. Awareness of the vulnerability of pregnant women and the ability to reduce the probability of infection were driving factors for vaccination.

‘We’re in contact with babies and pregnant women, who are more susceptible to experiencing complications from this illness. And I’m like a connecting bridge, you know? I can pass along the illness. So, I think it’s very important that I’m not the one to pass on the disease’.

(LL11)

### 3.3. Practices

#### 3.3.1. Informing, Recommending and Letting Pregnant Women Decide

Midwives offered varied responses when asked whether they recommended pregnant women to get vaccinated against influenza and pertussis, but they all agreed that the final decision belonged to the pregnant woman. Most participants reported that, rather than recommending vaccination, their practice was based on informing them, a distinction the midwives spontaneously expressed on their own. At the first visit, the pregnant woman was informed about the established vaccination period for pertussis and, if the visit took place at the time of the influenza vaccine campaign, the option of getting the shot the same day was offered. According to the providers, most women chose to get vaccinated for both illnesses.

‘I don’t advise them. I inform them and if they then request more information—I, like, “it’s up to you”. I explain what the science says. [...] And then they decide. [...] And if they have doubts, I tell them, “look, if you want, I’ll explain everything, you think it over, and you let me know at the next visit”. Then at the next visit they tell me, “hey, you mentioned the influenza vaccine”. I mean, I don’t even have to tell them. I have informed them, they’ve thought about and then they decide. [...] They all get vaccinated’.

(LL05)

Other participants described their practice as providing information about and recommending both vaccines. In such cases, the recommendation was very important and was not replaced by information. They upheld the notion that the final decision belonged to the woman.

‘At the first prenatal visit, you explain the tests you’ll do throughout the pregnancy. And you explain that both vaccines are recommended. [...] So I tell them, “you’re the one who makes the decision, but it is recommended that you get it”’.

(LL12)

Some midwives clearly stated their desire not to influence the pregnant woman’s decision: she had to be the one to decide. The professional had to limit herself to informing and recommending.

‘I don’t want to influence, no—I just don’t want to. [...] Because it’s an option they must have—It’s a recommendation. It’s not persuasion. It’s different. It’s a recommendation, that they must believe [...] Because they make their decision based on that. And it’s sort of the first decision. I always say, “this might be the first decision you have to make as a mother”’.

(LL16)

Lastly, some participants admitted to not recommending the influenza vaccine. The reasons included having little knowledge or information about the vaccine, doubts about its safety for the fetus, newborn and even the mother and a lack of evidence about its efficacy.

‘If this influenza vaccine ends up being neurotoxic to some woman, who has to deal with the side effects? She and her baby do, not me. So, until pharmaceutical companies offer more safety, I will have a hard time forcing women to—To campaign for them all to get vaccinated’.

(LL01)

#### 3.3.2. Semantics Matter: ‘It’s Recommended’ Versus ‘It’s Optional’

When the vaccine was explicitly recommended, the majority of midwives did so using the impersonal form (‘it’s recommended’) or ‘X institution recommends it’. Both options offer a more official and external picture. Only one midwife reported making the recommendation in the first person.

‘I tell them that the Government of Catalonia has started an influenza vaccination campaign. [...] I offer information about the campaign, tell them they’re in the risk group and that vaccination is recommended. And I offer to give them the shot. And most say, “ah, OK then”’.

(LL14)

‘At around week 28 or 29, I give them the information we have. I give them a brochure and tell them, “As you know we have this vaccine (pertussis). [...] I recommend you get it, because nothing I’m going to give you will affect the fetus at all, it offers benefits, comfort and protection for you and your child, ok?”’.

(LL09)

The idea of the vaccines being optional was part of the information that some midwives offered pregnant women, particularly for the influenza vaccine. Pregnant women were explicitly informed of their non-obligation and the optionality of getting vaccinated.

‘It’s explained to them that it’s optional, not obligatory, but that if they do get influenza, the consequences may be significant’.

(LL05)

However, some midwives pointed out that this openly offered optionality in the form of ‘you can get it’ or asking the pregnant woman if she wanted the vaccine, which were not conducive to her being vaccinated. Vaccination should not be imposed, but it should not be treated as optional either; it should be recommended.

‘I think it’s really important that you suggest it. Because it’s one thing to ask, “do you want to be vaccinated against influenza?” and another thing to say, “it’s recommended that you be vaccinated against influenza”. [...] I don’t tell them they’re obligated to get vaccinated and that if they don’t—No, no, no. I’m not patronizing either. No, no. I say it’s a recommendation and that as such I must convey it to them. And then I think that—Well, it has worked for me’.

(LL11)

#### 3.3.3. Role in the Event of Doubts or Requests for Advice: ‘What Would You Do?’

According to the midwives, pregnant women often raised doubts about the vaccines, particularly in the case of influenza. When this happened, participants mentioned providing information and facilitating the vaccination the same day as the visit or offering the option of doing it later, when the woman wanted. Among the midwives who recommended vaccination, some admitted to insisting less about the influenza vaccine than the others when pregnant women had doubts. The reasons for this included not wanting to convince women but rather allowing them to decide on their own, doubts about the side effects, and a lack of information. Others reported that, for the influenza vaccine, whether they insisted depended on the pregnant woman’s risk factors. This was not the case for pertussis.

‘Hmm. The one for tetanus/diphtheria and pertussis—I might insist more. [...] (Regarding the influenza vaccination) I review the medical history. If the girl, for example, has asthma or bronchitis, I insist. If the girl is healthy and has no associated factors, I say, “Think it over well, and remember that if you get influenza, you won’t be able to take anything for it. If you feel sure about that (not getting vaccinated), then do as you want”’.

(LL01)

When a pregnant woman requested advice and openly asked the midwife, ‘what would you do?’, there was a generally shared idea; midwives gave them the information or recommendations again, but not at the personal level, reinforcing the idea that it was the woman’s choice. The midwives conveyed not wanting to personally influence the woman’s final decision, despite recommending vaccination.

‘I recommend both, eh. And if they say, “what would you do, personally?” I always respond, “it’s a personal matter and I can’t influence your decision”’.

(LL08)

However, a small part of participants offered a non-nuanced response based on the benefits of vaccination. Moreover, they offered their own personal vaccination as an example. Insisting on the recommendation if the woman had doubts and convincing her to get vaccinated was part of their professional duty.

‘Some are hesitant, and I convince them. For the influenza vaccine, as well. The woman comes in, if it’s the influenza vaccine season, and asks me, “should I get it, X?”, I say, “yes, of course, you should get it. It’s protection that you take now for the entire pregnancy. Later on, I’ll give you the pertussis shot. Remember that we’re all in the same group. I also get the vaccine”. I mean, that’s my duty. I think it is’.

(LL09)

## 4. Discussion

Achieving the aim of determining the knowledge, perceptions, attitudes and practices of midwives regarding maternal influenza and pertussis vaccination using a qualitative methodology with semi-structured, face-to-face interviews makes this study unique, given how few publications have used this approach in Spain.

Our results demonstrate that midwives had different knowledge and perceptions about the two illnesses in pregnant women; they considered influenza less severe than pertussis for pregnant women at the same or slightly higher risk of contracting it than the rest of the population and believed that any complications resulting from influenza mostly affected the woman and not the fetus or newborn. In contrast, participants perceived pertussis to be very serious for newborns. According to previous studies, these results also extend to the pregnant women themselves. Understanding influenza as less serious, trivializing it even [27], not perceiving themselves at risk of contracting it [14,27] and understanding the severity as mostly affecting the mother and not the future child are all factors reported by pregnant women as influential in their final decision to get the influenza vaccine or not [14,23,27]. This leads to lower vaccination coverage for influenza than for pertussis. As far as the benefits of vaccination are concerned, the results are similar among pregnant women, who attribute greater protection to the mother in the case of the influenza vaccine and greater protection to the newborn in the case of the pertussis vaccine [24,26]. All this suggests a uniform understanding in the collective imagination of both groups, distinguishing between influenza and pertussis and the respective benefits of being vaccinated. This would seem logical given a population that receives information from healthcare providers, with midwives being the main source of professional advice among pregnant women [41,42], transmitting their own ideas and impacting the population they care for through their practices. These data indicate that addressing these professional ideas and practices first is a prerequisite for changing the view of pregnant women.

Regarding knowledge and perceptions of the safety of the vaccines, most participants trusted them, which coincides with other research studies showing that midwives did not have concerns about the safety of the vaccines [43]. That said, and in line with other studies, some participants had doubts, mainly with regard to the influenza vaccine, establishing side effects as the main barrier to recommendation [14], which was also found for midwives in Spain [21]. Despite evidence of vaccine safety [6,8,9,10], doubts about this issue still exist among professionals, which may once again have an impact on the persistence of these doubts among the population, as suggested by the same controversy appearing in studies with pregnant women [18,20]. A lack of information from institutions contributes to the doubts about safety that the participants expressed and which ultimately affected their recommendations. Not having enough knowledge or education about maternal vaccination comes up in various studies as a barrier to vaccine uptake [21,44,45]. The participants in this study, nurses–midwives, received such knowledge during their specialized training [46]. Nevertheless, they expressed a need for more information, which supports previous evidence showing that 97% of midwives need [43] and want [44] more information. This would suggest a need for ongoing education that helps keep professionals up to date about maternal immunization and vaccination, giving them the evidence they require to recommend vaccination. Once again, it would seem urgent to first address the professional sphere in order to produce a change in the population.

Both in this study and other pieces of research [43], midwives had a positive attitude toward vaccination. However, while all participants justified the pertussis vaccine and none spoke out against it, there was not as clear justification for the influenza vaccine due to doubts about the seriousness of the infection and the lower frequency of severe illness. This distinction was also identified in another study, in which 91.2% of midwives supported the pertussis vaccine, whereas only 79.2% supported the influenza vaccine [43]. As for midwives’ attitudes toward being vaccinated themselves, it is worth noting that most were not vaccinated against influenza in the current or previous campaigns. This is not surprising if we bear in mind that only 28% [47] of HCPs in Catalonia—where the data for this study were collected—were vaccinated. Vaccination figures from other contexts support these data, with less than 50% of nurses being vaccinated during the 2019–2020 season [48]. This figure is clearly below 75%, the achievable benchmark rate [49], demonstrating yet again the challenge of getting HCPs vaccinated. The little importance attributed to influenza is one of the reasons why professionals do not get vaccinated [50,51]. In the case of midwives, our results show that there was also a low perceived risk of contracting the illness due to lack of exposure to the virus since they worked with pregnant women, who were considered healthy patients. Only the midwives who had been vaccinated against influenza made reference to the vulnerability of pregnant women and wanting to reduce the likelihood of infecting them as a reason for being vaccinated as an HCP. Despite knowing that pregnant women represented a risk group, other participants did not find this vulnerability reason enough to get vaccinated and protect them. This fact was reflected in the widely held perceptions about the lack of severity and risk of influenza in pregnant women. A review of the studies conducted with nurses showed that the greater the knowledge and perception professionals had of risk, the greater the tendency to be vaccinated and recommend patients to do the same was [52]. It is crucial that all midwives, without exception, perceive pregnant women as a risk group, with the implications that entails, and perceive themselves as a potential source of transmission. Once again, one way of achieving this is by improving knowledge to help replace erroneous information with accurate data. Adjusting this perception must lead to an attitudinal change that is reflected in midwives’ own vaccination practices and the recommendations they make to pregnant women.

One key result of this study is the prevailing idea that the pregnant woman must make her own decisions. Respecting the rights of pregnant women in this regard is very important to midwives [45] and consistent with the desire to provide care focused on women’s needs and preferences [53]. It also falls in line with legislation on the right of all patients to freely make their own decision [54]. However, the professional role undertaken by many midwives is essentially informative [45], facilitating or that of impersonal adviser, since they do not want to influence pregnant women’s behavior in order to respect their autonomy. This role clashes with their professional responsibility to promote healthy behaviors, such as maternal vaccination. Consequently, this generates personal conflict for some midwives, as also conveyed in other studies [55]. On the other hand, it is also legally established that patients must make their own decisions after receiving appropriate information about the options available. Therefore, it is worth recalling existing evidence and international consensus on the need for maternal vaccination against influenza and pertussis [1,2,11,56]. Even so, midwives expressed that such evidence was not available to them and that they feared influencing a decision that might have negative consequences for pregnant women and newborns, all of which conditioned them not to recommend the influenza vaccine. Training, professional reflection and management actions must be implemented to reverse this fact. We must not forget that a recommendation from professionals is critical in pregnant women’s final decision [27].

The way in which professionals provide information and make recommendations is important [57] and portraying vaccination as optional, recommending it using the impersonal form (‘it is recommended’) or on an institutional basis influences the final behavior of pregnant women. Openly recommending vaccination, highlighting its benefits and insisting on it to achieve an attitudinal change that promotes maternal vaccination should be the norm among professionals. Not doing so results in the loss of an extraordinary opportunity. For this to happen, however, professionals’ attitudes towards maternal vaccination must be improved and reflected in their practices of recommending vaccines. Focusing on improving knowledge of vaccines and their benefits, working to eradicate false information about their safety, particularly in the case of influenza, and continuously offering professionals the scientific arguments and evidence available can help lead to this change.

One of the many limitations of inquiring after professional attitudes and practices is obtaining unrealistic answers with regards to what is expected professionally. However, face-to-face interviews conducted by experts, with appropriate questions and cross-examinations, and establishing an environment that promotes rapport all help minimize this inherent limitation to this kind of study. Another potential limitation is that the study population only covered Spain, where midwifery practices differ from those in other countries. However, the results are greatly needed in this context and can help in other contexts in which the midwife is responsible for monitoring pregnant women and making vaccination recommendations. Lastly, it should be noted that only professionals working in the public health system were included. Nevertheless, more midwives work in the public system than in private centers and thus attend to more pregnant women.

## 5. Conclusions

Most midwives have a positive understanding of and attitude toward maternal vaccination, considering it to be a safe strategy. However, doubts about the severity of influenza, lack of information about vaccine safety and the desire to play an informative professional role without influencing the decision of pregnant women result in less frequent recommendation and influenza vaccination coverage. Since midwives are one of the main sources of professional advice that pregnant women receive, it is urgent that their understanding and professional practices be addressed to change the attitude of pregnant women and thus increase vaccine uptake among them, particularly for influenza.

These results clearly have an impact on professional practice. Midwives need more information, ongoing training and evidence that allows them to recommend maternal vaccination straightforwardly and confidently, particularly in the case of influenza. They also require training on how to provide information and more effectively make vaccine recommendations. Such training should be guaranteed by academic and health institutions during their basic education and throughout their career as a means of bringing about a change in their conceptions, attitudes and practices.

The results also have policy implications since they can help refocus maternal vaccination strategies and programs, directly improving vaccination coverage for pregnant women, especially against influenza. Therefore, greater communication efforts should be made during vaccination campaigns to dispel misconceptions regarding the vaccination of pregnant women and adapt the information to the needs of this group. In the case of influenza vaccination, other political measures could be implemented, such as professional incentives based on midwives meeting certain targets. This strategy has been effective in the vaccination of pregnant women against pertussis.

## Figures and Tables

**Table 1:** Semi-structured interview guide.

Topics of Interest	Guiding Questions
Influenza vaccination as a professional	Can you explain the reasons why you did or did not get vaccinated this season?
	Did you get vaccinated in past seasons? Why did you or did you not decide to be vaccinated?
	Do you intend to get vaccinated in future seasons?
Knowledge and perceptions about influenza and pertussis in pregnant women	Can you explain what you know about influenza/pertussis in pregnant women?
	What do you think about severity of influenza/pertussis in pregnant women?
	* Supplementary questions: Do you think it is serious? For whom? How does it affect them?
Maternal vaccination against influenza/pertussis. Knowledge and perceptions of: - Benefits - Efficacy - Risk - Safety	What do you know about the influenza/pertussis vaccine in pregnant women?
	Can you tell me about the benefits of the influenza/pertussis vaccine while pregnant?
	* Supplementary questions: Do you think it is beneficial to get vaccinated against influenza/pertussis while pregnant? Which one? For whom?
	Can you tell me about the efficacy of the influenza/pertussis vaccine while pregnant?
	* Supplementary questions: Do you think influenza/pertussis vaccines are effective while pregnant?
	Can you tell me about the risk of the influenza/pertussis vaccine while pregnant?
	* Supplementary questions: Do you think there are any risks in being vaccinated against influenza/pertussis while pregnant? What are they? Do you think there are any risks in not being vaccinated?
	What do you think about the safety of the influenza/pertussis vaccine while pregnant?
Justification of vaccination (influenza/pertussis) in pregnant women	Do you think influenza/pertussis vaccination is justified in pregnant women?
Importance given to professional recommendations of vaccination (influenza/pertussis)	Do you think that the midwife’s recommendation has any weight in the final decision of pregnant women to get vaccinated or not? Why?
Vaccine recommendation (influenza/pertussis) in pregnant women	Do you recommend that pregnant women be vaccinated against influenza/pertussis?
	▪ Yes:
	What are the reasons for making this recommendation?
	Can you describe/give an example of how you make this recommendation?
	Can you explain what you do if a pregnant woman is not sure about it or does not want to be vaccinated?
	▪ No:
	What are the reasons for not making this recommendation?
	Can you describe/give me an example of what you explain to pregnant women about vaccination?
	Can you explain what you do if a pregnant woman is not sure about it or does not want to be vaccinated?

“*” Supplementary questions.

**Table 2:** Participants’ characteristics (*n* = 16).

Characteristic	Frequency
Gender	
Female	16
Male	0
Age (in years)	
25–30	1
31–40	5
41–50	5
51–60	2
61–65	3
Marital Status	
Single	2
Married	14
Number of children	
0	2
1	2
2	9
3	3
Experience as a midwife (in years)	
0–5	3
6–10	4
11–20	4
21–30	2
31–40	3
Vaccinated against influenza in the current season	
Yes	4
No	12
Ever vaccinated against influenza in previous seasons	
Yes	8
No	8

## Data Availability

The data that support the findings of this study are available on request from the corresponding author. The data are not publicly available due to privacy or ethical restrictions.
